# TGF-*β*2 levels in the aqueous humor are elevated in the second eye of high myopia within two weeks after sequential cataract surgery

**DOI:** 10.1038/s41598-022-22746-4

**Published:** 2022-10-26

**Authors:** Weijia Yan, Yaping Zhang, Junguo Cao, Hong Yan

**Affiliations:** 1 Shaanxi Eye Hospital, Xi’an People’s Hospital (Xi’an Fourth Hospital), Affiliated Northwestern Polytechnical University, Xi’an, 710004 Shaanxi China; 2 Department of Ophthalmology, University of Heidelberg, 69120 Heidelberg, Germany; 3 Division of Experimental Neurosurgery, Department of Neurosurgery, University of Heidelberg, 69120 Heidelberg, Germany

**Keywords:** Diseases, Risk factors

## Abstract

Transforming growth factor-*β* (TGF-*β*) is the critical regulator of physiological and pathological conditions in lens. The TGF-*β* signaling pathway is closely associated with high myopia patients. Thirty eyes from fifteen patients with high myopia who received sequential cataract surgery were enrolled in this prospective study. Ten cataract patients with non-myopia were chosen as a control group. Aqueous humor (AH) samples were used to detect the levels of TGF-*β*1, TGF-*β*2, and TGF-*β*3 in both groups. Compared with the non-myopic cataracts patient group, the highly myopic cataracts group had a significantly higher TGF-*β*2 (*P* = 0.019). Besides, the level of TGF-*β*2 of the second eye was significantly higher than that in the first eye in high myopia cataract patients group (*P* = 0.037). And TGF-*β*1 showed significant differences with age and axial length of high myopia cataract patients. Therefore, TGF-*β2* may contribute to the development of high myopia and cataract surgery increased the expression of TGF-*β2.*

## Introduction

Cataract surgery is one of the most common surgical procedures performed worldwide. Authoritative data show that the improvement in visual function and quality of life after binocular cataract surgery far exceeds that of monocular cataract surgery^1^. Therefore, timely second-eye surgery is essential for patients. In addition to routine cataract surgery, modern cataract surgery in highly myopia eyes has been shown to improve visual acuity. However, phacoemulsification-related surgery for high myopia patients is thought to be a risky procedure with common intraoperative and postoperative complications, such as posterior capsular rupture, retinal detachment, progressed myopic traction maculopathy, and capsular contraction syndrome^2^. In addition, high myopia is considered an inflammation-related disease^3,4^. Previous studies have demonstrated the expression of several inflammatory cytokines in the aqueous humor (AH) of high myopia^5^. For example, Zhang et al. tested 440 cytokines in the AH of eyes with high myopia cataract patients, and they found that MMP-2 and ANG-1 levels were significantly increased compared to non-myopic cataract patients^6^.

Transforming growth factor-*β* (TGF-*β*) is a pleiotropic cytokine involved in cell proliferation, differentiation, and apoptosis and also promotes extracellular matrix synthesis and wound healing. There are three TGF-*β* isoforms, TGF-*β*1, TGF-*β*2, and TGF-*β*3 in mammals, which play an important role in the regulation of physiological and pathological conditions in the lens^7^. Recently, studies showed that all three TGF-*β* isoforms contribute to the development of cataractous changes^8^. Our previous research identified higher level of TGF-*β*2 in the second eye than the first eye after measuring thirty-three cytokines in AH samples from both eyes of cataract patients after sequential cataract surgery^9^. Furthermore, elevated TGF-*β* also involved in scleral remodeling by accelerating epithelial–myofibroblast transdifferentiation in highly myopic eyes ^10,11^. For cataract patients, high myopia leads to a higher risk of developing ocular diseases and impair the vision of patients. However, the pathogenesis of the cataract patients with high myopia remains unclear.

As a follow up study of our published studies^1,9^, we evaluated the levels of TGF-*β* in both eyes of high myopic cataract patients after receiving sequential cataract surgery. Then, the expression levels of TGF-*β* in high myopia cataract patients was compared with non-myopia cataract patients. Next we compared the TGF-*β* levels between the first eyes and the second eyes in high myopia cataract patients. Besides, we analyzed the expression levels of TGF-*β* in different groups of axial length (AL), ages, and gender. Therefore, we aimed to clarify whether TGF-*β* contribute to the development of high myopia and the effect of cataract surgery on the expression of TGF-*β.*

## Materials and methods

### Selection of patients

This prospective longitudinal study was approved by the local ethics committee of Xi’an People’s Hospital, China (No 20200035). It was performed by the Declaration of Helsinki. Written informed consent was obtained from all patients. From Jan. 2020 to July 2020, AH samples were collected from 15 patients with high myopia cataracts. AL was measured with an ophthalmology ultrasound system (IOLMaster, Zeiss, Germany). The systematic ophthalmological examinations were performed during the pre-surgical visit to exclude patients with a history of eye surgery or any known eye diseases, such as glaucoma, retinitis, uveitis, and progressive retinal diseases. Patients who received local medications or underwent laser treatment and complications from the intra-operative were also excluded. From the day of admission to two weeks after surgery, all patients were asked to use 0.3% levofloxacin. All cataract surgeries were performed by the same surgeon (YH) under topical anesthesia using phacoemulsification with intraocular lens implantation.

### Aqueous humor sample collection

Samples were obtained at the beginning of clear lens extraction or cataract extraction surgery. A 27-gauge needle was used to aspirate AH samples (100–200 μl) from the central pupillary area without touching the iris, lens, or corneal endothelium to avoid trauma and contamination of AH samples. Specimens were placed inside sterile Eppendorf tubes that were immediately stored below − 80 °C until analysis.

### Analysis of TGF-β1, TGF-β2, and TGF-β3 concentrations in AH samples

All analytical procedures were performed according to the manufacturer’s instructions. Proprietary techniques were used to internally color-code microspheres with two fluorescent dyes. After concentrating the dyes, each dye was coated with a specific capture antibody. Next, an analysis from a test sample was captured by the bead to introduce the detection antibody. A Luminex® analyzer (MAGPIX®) was used to identify each microsphere, and the result of its bioassay was quantified based on fluorescent reporter signals. EMD Millipore combines the streamlined data acquisition power of Luminex® xPONENT® acquisition software with the sophisticated analytic capabilities of the new MILLIPLEX® Analyst 5.1, integrating data acquisition and analysis seamlessly with all Luminex® instruments.

### Statistical analysis

All data are mean ± standard deviations. The χ2 tests was used to examine differences in categorical variables. Shapiro–Wilk tests were employed to assess whether the data were normally distributed. If the criteria for normal distribution were satisfied, an unpaired/paired *t*-test was used for comparison; if not, the nonparametric Mann–Whitney-U-test was adopted. The student’s *t*-test was used to detect differences between the first- and second eye surgery groups. unpaired *t*-tests were used to detect differences between the high myopia cataract patients and non-myopia cataract patients. The linear regression test was used to analyze the relationship between TGF-*β*1, TGF-*β*2, and TGF-*β*3 concentrations and AL, ages, and gender. The results of each statistical test were shown in Supplementary Table 1. All analyses were performed using GraphPad Prism software version 8.0 (GraphPad Software, San Diego, CA). A two-tailed *P* < 0.05 was considered statistically significant for this analysis.

## Results

### Baseline characteristics of all cataract patients

The study comprised a total of 30 eyes (15 patients) who satisfied the inclusion criteria. Demographic characteristics of the patients are present in Table 1 and Table 2. There were no statistically significant differences among the cataract (control) group and surgery eyes group in terms of age and sex. And the two surgery eyes group differences in operated eye, AL and ACD were not statistically significant (all *P* > 0.05, unpaired/paired *t*-test for age, AL and ACD; χ2 tests for sex and eye[s] operated on). Table 3 shows the statistics of best-corrected visual acuity, the visual acuity without correction, and spherical equivalent diopter of both eyes during the preoperative time and 1 week and 1 month after surgery.

**Table 1:** Baseline characteristics of cataract patients and high myopia cataract patients.

	Cataract	High myopia cataract	*P* Value
Age, Mean ± SD (year)	60.9 ± 11.6	53.9 ± 8.9	0.114*
No. of males/females	3/7	5/10	0.861^†^

*Shapiro–Wilk test and Unpaired *t*-test.

^†^χ2 test.

**Table 2:** Baseline characteristics of patients between the two surgery eyes.

	First eye	Second eye	*P* Value
No. of operated eyes, right eye/left eye	10/5	5/10	0.068*
AL (mm)	30.63 ± 2.50	30.17 ± 2.76	0.206^†^
AC depth (mm)	3.51 ± 0.44	3.54 ± 0.44	0.347^†^

*AL* length of axial; *AC* anterior chamber.

*χ2 tests.

^†^Shapiro–Wilk test and Paired *t*-test.

**Table 3:** Pre- and postoperative values of the two surgery eyes.

First-eye	Preoperative	Postoperative(1w)	Postoperative(1 m)
VAsc	1.63 ± 0.46	0.54 ± 0.38	0.48 ± 0.44
BCVA	0.91 ± 0.56	0.27 ± 0.41	0.23 ± 0.49
SE (diopter)	− 19.92 ± 5.49	− 2.13 ± 0.90	− 1.90 ± 0.81

Second-eye	Preoperative	Postoperative(1w)	Postoperative(1 m)
VAsc	1.43 ± 0.48	0.50 ± 0.18	0.40 ± 0.21
BCVA	0.69 ± 0.34	0.18 ± 0.19	0.18 ± 0.20
SE (diopter)	− 18.17 ± 4.04	− 2.57 ± 0.64	− 2.32 ± 0.54

*BCVA* best corrected visual acuity (LogMAR); *VAsc* visual acuity without correction (LogMAR); *SE* spherical equivalent.

### Concentrations of TGF-β1, TGF-β2, and TGF-β3

The comparison of TGF-*β*1, TGF-*β*2, and TGF-*β*3 concentrations in AH between high myopia cataract patients and non-myopia cataract patients, and the first and second operated eyes are summarized in Table 4. The concentration of TGF-*β*2 in high myopia cataract patients was significantly greater than in cataract patients (*P* = 0.019), whereas TGF-*β*1 and TGF-*β*3 concentrations were not significantly different between these two groups (*P* = 0.509, *P* = 0.985). Furthermore, the level of TGF-*β*2 was significantly higher in the second operated eye than in the first operated eye (*P* = 0.037). However, the levels of TGF-*β*1 (*P* = 0.324) and TGF-*β*3 (*P* = 0.76) did not exhibit significant differences between these two groups of eyes.

**Table 4:** The expression levels of TGF-*β*1, TGF-*β*2 and TGF-*β*3 in the cataract and high myopia cataract patients.

	High myopia cataract patients		*P* Value	Cataract and High myopia cataract patients		*P* Value
	Cataract	High myopia cataract		First eye	Second eye	
TGF-*β*1 (pg/ml)	33.01 ± 13.42	42.36 ± 21.38	0.509*	36.64 ± 13.51	39.71 ± 21.14	0.992*
TGF-*β*2 (pg/ml)	3052.11 ± 661.10	3924.73 ± 856.69	0.019^†^	3346.67 ± 589.04	3924.73 ± 856.69	0.037^‡^
TGF-*β*3 (pg/ml)	24.25 ± 8.61	24.18 ± 8.85	0.985^†^	23.21 ± 6.97	24.18 ± 8.85	0.769^‡^

Values are concentrations of the TGF-*β*, means ± SD.

*Shapiro–Wilk test and Mann–Whitney-U-test.

^†^Shapiro–Wilk test and Unpaired t-test.

^‡^Shapiro–Wilk test and paired *t*-test.

### Relationship between TGF-β1, TGF-β2, and TGF-β3 and axial length

**Figure 1:**
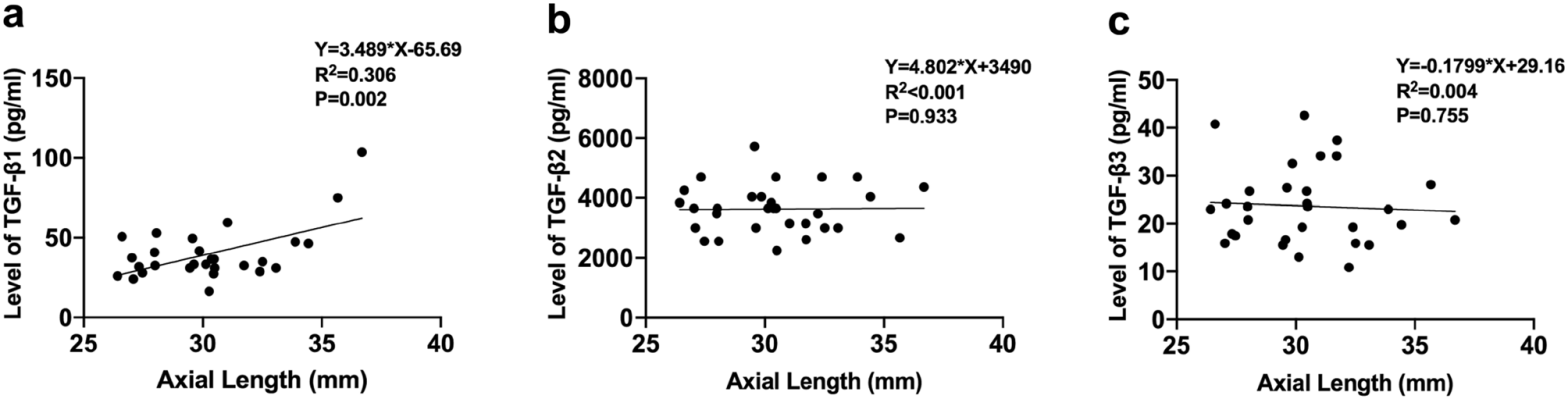
Scatter plots show the relationships between the concentration of TGF-*β*1 (**a**), TGF-*β*2 (**b**), and TGF-*β*3 (**c**) with AL.

To further investigate the relation between AL and the level of TGF-*β*s*,* 30 AH samples from high myopia cataract patients were used. The median levels in AH were 38.18 ± 17.81 pg/ml (mean ± standard deviation) TGF-*β*1, 3635.7 ± 790.32 pg/ml TGF-*β*2, and 23.69 ± 7.98 pg/ml TGF-*β*3. Linear regression analysis showed significant differences among AL in terms of the concentration of TGF-*β*1 (*R*^2^ = 0.306, *P* = 0.002) (Fig. 1a), but no significant differences were detected between the concentrations of TGF-*β*2 (Fig. 1b) and TGF-*β*3 (Fig. 1c).

### Relationship between TGF-β1, TGF-β2, and TGF-β3 and age

**Figure 2:**
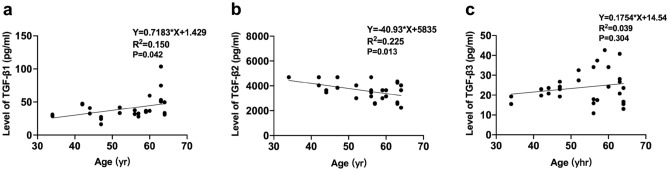
Scatter plots show the relationships between the concentration of TGF-*β*1 (**a**), TGF-*β*2 (**b**), and TGF-*β*3 (**c**) with AL.

The aqueous humor TGF-*β*1 concentration was positively correlated with age (*R*^2^ = 0.150, *P* = 0.042) (Fig. 2a), while the TGF-*β*2 concentration decreased with age (*R*^2^ = 0.225, *P* = 0.013) (Fig. 2b). No relationship between the concentration of TGF-*β*3 and age was found (*R*^2^ = 0.039, *P* = 0.304) (Fig. 2c).

### Relationship between TGF-β1, TGF-β2, and TGF-β3 and gender

In our study, there were no significant differences in TGF-*β*1, TGF-*β*2, and TGF-*β*3 between females and males (*P* = 0.145, *P* = 0.353, *P* = 0.287) (Table5).

**Table 5:** The expression levels of TGF-*β*1, TGF-*β*2 and TGF-*β*3 in male and female high myopia cataract patients.

	Male	Female	*P* Value
TGF-*β*1 (pg/ml)	40.83 ± 9.17	36.85 ± 20.69	0.145*
TGF-*β*2 (pg/ml)	3832.30 ± 873.46	3537.40 ± 725.54	0.353^†^
TGF-*β*3 (pg/ml)	21.42 ± 5.58	24.83 ± 8.73	0.287^†^

*Shapiro–Wilk test and Mann–Whitney-U-test.

^†^Shapiro–Wilk test and Unpaired *t*-test.

## Discussion

In the present study, we identified elevated TGF-*β*2 levels in high myopic cataract patients. The results showed that the levels of TGF-*β*2 were significantly increased in patients undergoing cataract surgery with high myopia on the second eye than those on the first eye. Meanwhile, the level of TGF-*β*2 was positively higher in high myopia cataract patients than in non-myopia cataract patients. TGF-*β*1 concentration in the AH was positively correlated with AL and age, on the other hand, the level of TGF-*β*2 decreased with age. We did not find a significant correlation between the levels of TGF-*β*1*,* TGF-*β*2*, and* TGF-*β*3 with gender.

TGF-*β*2 is an important factor in modulating the growth and development of the eyeball^12^. Animal models demonstrated that the sclera level of TGF-β2 protein was highly correlated with the high myopia^13,14^. In addition, clinical researches have shown that the concentration of TGF-*β*2 in AH is more elevated in high myopia cataract patients than in non-myopia cataract patients^11,15^. However, there are inconsistencies in the TGF-*β*2 expression during myopia formation as some studies have reported a decrease or do not significantly change^13,16,17^. It appears that the TGF- *β*2 expression alterations during the development of myopia appear to be species and tissue specific^18^. Our study found that TGF-*β*2 levels were increased in AH sample in high myopia patients compared to non-myopia patients. However, although TGF-*β*2 is a key factor in the progression of high myopia development, it is still unclear what role TGF-*β*2 plays in the process.

Interestingly, our study showed that the TGF-*β*2 concentration was more significant in the second eye compared with the first eye in high myopia cataract patients. Because the microenvironment conditions of the second eye can be affected by cataract surgery in the first eye^19^. Meanwhile, the TGF- *β* family is an important tissue repair mediator that shows a distinct pattern during normal acute wound healing^20^. According to Thompson et al., surgery-induced epithelium damage could increase TGF-*β*2 levels twofold^21^, therefore the higher TGF-*β*2 levels in the second operated eye could represent a response to the first eye operation. In addition, our previous study found a higher level of TGF-*β*2 in the second cataract surgery eye compared to the first surgery eye^9^. Both of our studies identified that cataract surgery increased the level of TGF-*β*2 in cataract patients and high myopia cataract patients. Furthermore, we compared the extent of increase of TGF-*β*2 in both groups of patients. For cataract patients, the level of TGF-*β*2 in the second eye increased by 23.49%, from 2419.84 pg/ml to 2988.19 pg/ml. For high myopia cataract patients, the level of TGF-*β*2 in the second eye increased by 17.27%, from 3346.67 pg/ml to 3924.73 pg/ml. Although the increase rate of high myopia cataract patients was lower than cataract patients, which may contribute to that high myopia patients already shown a higher level of TGF-*β*2. However, high level of TGF-*β*2 may increase the risk of capsular contraction syndrome (CCS) and posterior capsular opacification (PCO), which are the complication of cataract surgery^18^. Further research is needed to investigate whether TGF- *β*2 inhibitors could decrease the risk of these complications.

The relationship between the level of TGF- *β* and AL has been compared in numerous in vivo and in vitro studies^22,23^. In a chick model, Rohrer and Stell observed that TGF-*β*1 was a potent inhibitor of basic fibroblast growth factor, which restrains the progression of myopia^22^. Furthermore, clinical investigation, Haiso et al. found that TGF-*β*1 was increased in eyes with excessive elongation of AL, which was consistent with our results^23^. Some studies have also shown a relationship between TGF-*β*2 and AL. For example, Jia et al. reported that the concentrations of TGF-*β*2 in 65 patients were positively correlated with AL^15^. In our study, we did not find a significant correlation between the level of TGF-*β*2 and AL. However, the AL in that study ranged from 24 to 29 mm, while our subjects ranged from 26 to 34 mm. In addition to the AL, we discovered substantial variations in TGF-*β*1 and TGF-*β*2 levels with age, with TGF- *β*2 being adversely linked with age. Studies on glaucoma patients reported that the TGF-*β*2 concentration in AH decreased with age^24,25^. The cause is thought to be the decreased ability with age of the tissues to produce TGF-*β*2^24^.

In conclusion, the results of the present study revealed that the concentration of TGF-*β*2 in the AH samples was significantly higher in the second surgery eye than in the first surgery eye in high myopia cataract patients, and the high myopia group had significantly higher TGF-*β*2 concentration than non-myopia cataract group. This finding suggests that eyes with high myopia may have a special internal microenvironment and TGF-*β*2 may contribute to the development of high myopia. Meanwhile, it has been recommended that during binocular surgery for high myopia cataract patients, the contralateral eye should be adequately delayed for surgery time. Although it may cause problems of binocular balance, it might be able to avoid the complications caused by the increase in TGF-*β*2 concentration caused by short-term second-eye surgery.

## Supplementary Information

Supplementary Information.

## Data Availability

The datasets used and/or analyzed during the current study are available from the corresponding author on reasonable request.
