# Supplementary material for: TGF-β2 levels in the aqueous humor are elevated in the second eye of high myopia within two weeks after sequential cataract surgery
Source: Sci Rep. 2022 Oct 26;12:17974. doi: 10.1038/s41598-022-22746-4 (PMC9606117; doi:10.1038/s41598-022-22746-4)
Supplement: Supplementary file 1 — Supplementary Information. [file 41598_2022_22746_MOESM1_ESM.docx]

Supplemental Table 1. Statistical testes and P value for each figure

| Figure 1 | Cataract | Myopia and Cataract | Methods | *P* value |
| --- | --- | --- | --- | --- |
| TGF-*β*1 | Yes | No | Mann-Whitney-U-test | 0.509 |
| TGF-*β*2 | Yes | Yes | Unpaired t-test | 0.019 |
| TGF-*β*3 | Yes | Yes | Unpaired t-test | 0.985 |

| Figure 2 | First eye | Second eye | Methods | *P* value |
| --- | --- | --- | --- | --- |
| TGF-*β*1 | Yes | No | Mann-Whitney-U-test | 0.992 |
| TGF-*β*2 | Yes | Yes | paired t-test | 0.037 |
| TGF-*β*3 | Yes | Yes | paired t-test | 0.769 |

| Figure 3 | AL < 31 mm | AL ≥ 31 mm | Methods | *P* value |
| --- | --- | --- | --- | --- |
| TGF-*β*1 | Yes | Yes | Unpaired t-test | 0.017 |
| TGF-*β*2 | Yes | Yes | Unpaired t-test | 0.822 |
| TGF-*β*3 | Yes | Yes | Unpaired t-test | 0.936 |

| Figure 4 | Age < 60 | Age ≥ 60 | Methods | *P* value |
| --- | --- | --- | --- | --- |
| TGF-*β*1 | Yes | Yes | Unpaired t-test | 0.001 |
| TGF-*β*2 | Yes | Yes | Unpaired t-test | 0.204 |
| TGF-*β*3 | Yes | Yes | Unpaired t-test | 0.759 |

| Figure 5 | Female | Male | Methods | *P* value |
| --- | --- | --- | --- | --- |
| TGF-*β*1 | No | Yes | Mann-Whitney-U-test | 0.145 |
| TGF-*β*2 | Yes | Yes | Unpaired t-test | 0.353 |
| TGF-*β*3 | Yes | Yes | Unpaired t-test | 0.287 |

Yes: means the data was normally distributed;

No: means the data was not normally distributed
